# Psychological Predictors for Depression and Burnout Among German Junior Elite Athletes

**DOI:** 10.3389/fpsyg.2020.00601

**Published:** 2020-04-02

**Authors:** Insa Nixdorf, Jürgen Beckmann, Raphael Nixdorf

**Affiliations:** ^1^Chair of Sport Psychology, Department of Sport and Health Sciences, Technical University of Munich, Munich, Germany; ^2^School of Human Movement and Nutrition Sciences, The University of Queensland, St Lucia, QLD, Australia; ^3^Physical Education and Sport Sciences, The University of Limerick, Limerick, Ireland

**Keywords:** depression, diathesis, vulnerability, longitudinal study, athlete burnout, stress

## Abstract

There exists a strong need for research in clinical sport psychology which does not merely gather information on prevalence rates for psychological disorders and case studies of affected athletes. Rather, research should also uncover the underlying psychological variables which increase the risk for depression and burnout in elite athletes. Many studies gather general factors (e.g., gender, injury, sport discipline) and stay on a more descriptive level. Both constructs (burnout and depression) are based on a temporal, stress-related process model assuming the development of either syndrome results from unfavorable personal (e.g., dysfunctional attitudes, perfectionism, negative coping strategies) or environmental (e.g., cohesion) factors coexisting with severe stressors (i.e., chronic stress). Integrating this knowledge, we propose a shared model for depression and burnout in athletes: a sport specific diathesis-stress model. The present longitudinal study assesses data throughout one sporting season to analyze predictors for both constructs in junior elite athletes. Hierarchical multiple linear regression analyses resulted in six predictors for best model fit. The following factors demonstrated a significant impact on predicting (a) burnout or (b) depression scores at the end of the season: dysfunctional attitudes (a and b), coping strategies (a and b), perfectionism (a), recovery (b), stress (a) and the level of depression at onset (b). Variables such as cohesion or attributional style did not significantly predict depression or burnout. The study supports the structure of a process model (diathesis-stress model) for burnout and depression with the assumption of temporal progression. With some vulnerabilities and their temporal, developmental link identified, prevention can become athlete-specific, effective and economical.

## Introduction

Postulate number 10 of the International Society of Sport Psychology position stand states:

Based on the analysis of mental health research and practice, the International Society of Sport Psychology sets the following challenges for sport psychology researchers and practitioners: (a) to further develop existing lines of research on various forms of athletes’ mental ill-being, including data on prevalence of mental illness, their sources, and forms of prevention and treatment (Schinke et al., 2017).

Depression and burnout are relevant syndromes in the context of elite sports. Empirical studies on prevalence rates for depression among athletes illustrate the potentially high risk for athletes to suffer from symptoms of depression (Wolanin et al., 2015). Depression is recognized as a psychological disorder characterized by symptoms of depressed mood, anhedonia, fatigue, and feelings of guilt (American Psychiatric Association, 2013). Furthermore, depression can be regarded as a multisystem disorder with affective, cognitive and physiological manifestations with serious problems including suicidal ideation (Lee et al., 2010; Hawton et al., 2013). In sport, this psychological disorder is also a considerable challenge. Depending on the sample and the assessment method, prevalence rates appear to be at a concerning level, averaging around 20% (Nixdorf et al., 2013; Gulliver et al., 2015; Wolanin et al., 2016). However, there is little evidence pointing out underlying mechanisms and factors which would help preventing syndromes of depression in sports (Frank et al., 2013). Longitudinal studies testing and exploring predicting factors for depression are necessary to capture such mechanisms.

Research on athletes’ burnout has investigated associated factors for over a decade (Goodger et al., 2007; Eklund and DeFreese, 2015), including longitudinal studies on its development (Isoard-Gautheu et al., 2015; Madigan et al., 2016; Martinent et al., 2016). However, its relation to other syndromes is still vague. In addition, the clinical relevance of (athlete) burnout is often criticized, with no agreement on a formal diagnosis, no prevalence rates can be established (Bianchi et al., 2015a; Gustafsson et al., 2017). Athlete Burnout is commonly recognized as a three-dimensional syndrome involving physical and emotional exhaustion, sport devaluation and a reduced sense of accomplishment (Raedeke and Smith, 2001; Gustafsson et al., 2017). Due to its overlapping symptoms, there is an ongoing debate on the relation between burnout and depression (Bahlmann et al., 2013; Bianchi et al., 2013, 2015a,b). Many studies indicated that there are differences between the syndromes (Bakker et al., 2000; Toker and Biron, 2012; De Francisco et al., 2016). But its medical recognition is not fully established which makes it even harder to separate both constructs. However, research on both syndromes would help relating both constructs and could contribute to the ongoing debate. Thus, combining depression and burnout in a longitudinal study for deepening the understanding of the mechanism underlying these syndromes is our main goal with the present study.

Thus far, only some studies clearly address both concepts (e.g., De Francisco et al., 2016; Frank et al., 2017; Gerber et al., 2018a; Smith et al., 2018). These studies point out that, while symptoms of both may overlap, both syndromes only have little relation over time and the two concepts are not interchangeable (Frank et al., 2017). However, both constructs can be regarded from a stress perspective (De Francisco et al., 2016) and share to some degree stress-related prospective features (Gerber et al., 2018a). Consequently, further studies on both concepts with the aim of revealing and comparing underlying mechanisms might be highly valuable.

There are different approaches to studying burnout and depression in elite athletes. Recent research on depression tends to focus on general factors and explore symptoms on superficial differences. This research discusses broad factors such as injuries (Gouttebarge et al., 2015a), gender (Junge and Feddermann-Demont, 2016), and sport discipline (Schaal et al., 2011; Nixdorf et al., 2013). However, Nixdorf et al. (2016) indicate that focusing on identifying underlying mechanisms could contribute to a more detailed understanding and better prevention strategies. In contrast, recent research on athlete burnout is far more detailed and driven by theoretical assumptions (Eklund and DeFreese, 2015). Conceptualization of athlete’s burnout often includes assumptions about etiological processes. For instance, motivational aspects from the self-determination theory (Ryan and Deci, 2000) have often been utilized and shown valuable aspects in explaining athlete burnout (Madigan et al., 2016). Furthermore, there are suppositions on social explanations (Coakley, 1992) regarding athlete’s identities or process based models on stress (Smith, 1986).

In order to provide a useful overview, both constructs are highlighted regarding their recent findings. The focus therefore is on repeated findings or factors with promising associations to the constructs with regard to their potential underlying mechanisms. These factors are categorized as either personal or environmental.

### Personal Factors

#### Perfectionism

Perfectionism is a cognitive factor, which appears highly important for athlete’s burnout (Eklund and DeFreese, 2015). Perfectionism can be regarded as a personal disposition characterized by striving for flawlessness and setting exceedingly high standards. It is accompanied by overly critical evaluations of one’s behavior (see Frost et al., 1990; Hewitt and Flett, 1991b; Flett and Hewitt, 2002). The concept of a multidimensional personality disposition (Enns and Cox, 2002) has different aspects. Some aspects are regarded as maladaptive and other aspects as adaptive (Stoeber and Otto, 2006). Especially in athletes, research is concerned with the discussion about adaptive and maladaptive perfectionism (Gotwals et al., 2012). On the maladaptive side, perfectionistic concerns have been repeatedly linked to burnout in athletes (Hill et al., 2008; e.g., Hill, 2013; Madigan et al., 2015). Perfectionistic concerns can, for example, be perfectionistic expectations from others, coaches, teammates, and parents or negative reactions after imperfections (Enns and Cox, 2002; Stoeber et al., 2004). Longitudinal studies have supported the connection between perfectionistic concerns and athlete burnout (Madigan et al., 2015, 2016). In a cross-sectional analysis, a correlation between perfectionistic expectations from outside the individual athlete, a sub-category of perfectionism, and depressive symptoms was found in junior elite athletes (Nixdorf et al., 2016). Additionally, recent short-term longitudinal study indicated associations over time between socially prescribed perfectionism and symptoms of burnout as well as depression (Smith et al., 2018). Therefore, perfectionism could be in important predictor for burnout and depression in elite athletes.

#### Dysfunctional Attitudes and Attributional Style

In research on depression, negative cognition has been a major part of explaining underlying mechanisms in development and manifestation (Beck, 2005). Therefore, the concepts of dysfunctional attitudes and attribution have been of particular interest in clinical patients (e.g., Beck, 1967; Weissman and Beck, 1978; Hull and Mendolia, 1991; Kim-Spoon et al., 2012). Beck‘s cognitive model (Beck, 1974) posits that people are depressed because their thoughts and conclusions are subjected to negative distortions. Such distortions can be characterized, for example by arbitrary conclusions, selective abstraction, overgeneralization and over- or understatements. Distortions may be due to negative schemas or dysfunctional assumptions. Research showed that specific attitudes and beliefs (such as distorted or negative thinking, overgeneralized assumptions, or selective information processing) are apparent in depressed patients, thus concluding that such dysfunctional attitudes illustrate a vulnerability for clinical disorders such as depression (Brown and Beck, 2002; Beck, 2005).

Perfectionistic thinking is closely related to dysfunctional attitudes (Ashby and Rice, 2002). Further, perfectionistic thinking can be even considered a type of dysfunctional attitudes (Beevers et al., 2007; de Graaf et al., 2009). Because of an ongoing assessment and evaluation of performance, especially in competitive sports, an identification of possible negative distortions and dysfunctional attitudes is particularly important. This is also a critical point of discussion in relation to the function of perfectionism in sport, where exceedingly high standards might be desired for personal peak performance. Thus adaptive and maladaptive aspects should be considered in this domain (Gotwals et al., 2012). However, the concept of perfectionism is well known with regard to depression; research highlights the potential impact of perfectionistic attitudes (Hewitt and Flett, 1991a, 1993; Hewitt et al., 1996; Cox and Enns, 2003). Similar effects regarding to dysfunctional attitudes and mental problems could be assumed but have not been investigated in elite sports.

Empirical evidence indicated importance of attribution style in elite sports. Previously, studies showed higher depression scores in athletes of individual sport disciplines compared to team sport athletes. One psychological difference between team and individual sports may lie in the aforementioned negative distortions attribution of failure and success. Hanrahan and Cerin (2009) showed that athletes in individual and team sports differ in their style of attribution. As the authors point out, individual sport athletes might make more internal attributions without teammates to credit or blame for results. For positive events, this style of attribution holds potential benefits with regard to performance or persistence (Hanrahan and Biddle, 2008). On the other hand, for negative events it can be a risk factor for depression and negative mood (Abramson et al., 1989). Internal attribution after negative events (e.g., failure) is associated with negative affect, such as guilt and shame (Tracy and Robins, 2004). Nixdorf et al. (2016) showed that attribution after failure mediates the association between individual sport athletes and depression scores. Thus, attribution seems to play an important role in explaining the differential vulnerability to depression in team versus individual sports and further might be considered a risk factor for depression and burnout in sports in general.

#### Coping

Coping is considered to be a personal skill or a group of strategies used to handle stress and deal with negative events (Schmidt and Caspar, 2009). Wingenfeld et al. (2009) pointed to a significant difference in the use of coping strategies among healthy and depressive individuals. With regards to the high level of (chronic) stress elite athletes experience, coping strategies, in addition to excellent motor and sporting skills, are vitally important to a successful athletic career and even correlate with performance satisfaction (Nicholls et al., 2012). On the other side, negative or absent coping strategies might affect psychological problems resulting from exceeding levels of stress. Lending support to this hypothesis, Nixdorf et al. (2013) as well as Crocker and Graham (1995) show correlations between coping strategies and depressive symptomatology. Nixdorf et al. (2013) more specifically showed that the frequent use of negative coping strategies (e.g., escape, resignation, and self-pity) correlated with high levels of depressive symptomatology, whereas positive strategies (e.g., situation control and addressing oneself in encouraging tones) showed correlations with low levels of depression.

In the context of burnout, stress is an integral factor for the physical and emotional exhaustion characteristic of burnout, especially with occupational stress being regarded as a trigger for burnout (Maslach and Jackson, 1981; Maslach et al., 2001). Thus, strategies of coping with occupational stress should be important. In elite sports, early concepts for example by Smith (1986) were pointing out the importance of athlete coping resources to manage the stress involved in elite sports. Empirical evidence testing this assumption found coping to be important for the stress-burnout connection (Raedeke and Smith, 2004). Goodger et al. (2007) found in their review that, of six studies all showed negative associations between burnout and coping in athletes.

### Environmental Factors

#### Cohesion

Besides cognitive factors such as attribution or attitudes, social factors (cohesion or social support) are associated with depressive symptoms and their development (Alloy et al., 2006; Au et al., 2009). Therefore, low social support is associated with elevated depression scores. The relevance of these social factors for depression has been demonstrated for athlete, too (Armstrong and Oomen-Early, 2009; Ohlert, 2012). Recent articles indicated that, even in retired athletes, low social support relates to depression throughout and after the athlete’s career (Gouttebarge et al., 2015b). Further, recent results showed cohesion in teams and training groups to be negatively associated with depression in athletes (Nixdorf et al., 2016).

Research also highlighted the importance of social factors for athlete burnout. DeFreese and Smith (2014) conducted a four-wave longitudinal study assessing social factors such as social support and negative interactions in relation to burnout. When controlling for other relevant factors such as stress, results showed negative associations between athletes’ experiences of social support and burnout as well as positive associations between negative social interactions and burnout. In detail, the strongest associations were found for the dimension of emotional and physical exhaustion.

#### Recovery

In order to win competitions, achieve goals, and improve performances, athletes have to push themselves more and more toward their limits. An increase in exercise loads connected with physical and psychological stress tends to be common in professional athletes’ training schedule. Yet with increasing exercise loads, recovery becomes more important for athletes’ well-beings, an aspect that is often overlooked. Recovery can be described as an inter- and intra-individual process that occurs over time for the reestablishment of performance abilities (Kellmann, 2002). This process, which includes psychological, physiological, and social components, varies by person and situation, and underlies intentional regulations. Beckmann (2002) describes recovery as a process of self-regulation which should achieve detachment from a past activity followed by engagement in a new activity. The author argues, that it is important to fully deactivate the stressful activity because of the complex relationship between recovery and stress. If detachment from a stressful activity fails, recovery can be impaired, and an imbalance arises. Kellmann (2010) further argues that an imbalance between stress and recovery can lead to greater experiences of stress and eventually lead to illness, depression, burnout, or overtraining.

Focusing on the outcomes of depression and burnout there is some research highlighting the importance of recovery or the negative impacts of insufficient recovery. Various studies have demonstrated the association of high training loads with psychological symptoms, such as changes in tension, depression, anger, vigor, fatigue, and mood (Morgan et al., 1987, 1988; O’Connor et al., 1989; Raglin et al., 1991). This led researches to discuss the relevance of heavy training loads and symptoms of overtraining to depressive syndromes (Puffer and McShane, 1992; Armstrong and Van Heest, 2002). On an empirical level Nixdorf et al. (2013) found negative stress-recovery states characterized by high scores in stress and low scores in recovery to correlate with high levels of depressive symptomatology. This pattern was found for sport-related and for general stress-recovery states highlighting the possible protective role of recovery for depressive syndromes in elite athletes.

Apart from depressive symptoms, experiences of burnout are also recognized as a result of exercise stress and a lack of recovery (Lemyre et al., 2007). According to Angeli et al. (2004) burnout is a stress-related condition that consists of alteration of physiological functions and adaptation to performance, impairment of psychological processing, immunological dysfunction and biochemical abnormalities. Other researchers consider burnout as a stress-related syndrome (Smith, 1986; Gustafsson et al., 2011), in which exercise stress might play an important role. Goodger et al. (2007) found support for the association between burnout and training loads or a lack of recovery.

#### Stress

Whether one takes into consideration the important tournaments and potential sporting injuries (acute stress) or the frequency of tournaments and training sessions (chronic stress), the life of an athlete can be regarded as “stressful.” Based on the overall amount of time spent for training and practice, the level of demand for junior athletes increased by almost 25% between 1979 and 2000 (Fessler et al., 2002). In addition to the time spent on training, research on stressors in the context of sports shows that stressors can be found in the competitive surroundings as well as in the organization an athlete is located in (Hanton et al., 2005). Further, athletes are also exposed to other stressors such as difficulties balancing sport, study commitments (Noblet and Gifford, 2002) and the physical demand of training (Gould et al., 1993).

Consequently, stress-related mental health problems are expected and studies support assumptions that the amount of daily stress in athlete’s lives is sufficient to cause an essential burden (e.g., Gould et al., 1993; Puente-Diaz and Anshel, 2005; Reeves et al., 2009). Nixdorf et al. (2013) also found support for this assumption, indicating high associations between chronic stress and depressive syndromes, as well as between different coping strategies and depressive syndromes in elite athletes. Nixdorf et al. (2015) showed that athletes with major stressors regarding psychological and physiological challenges in the direct context of their sport (e.g., heavy exercise loads, psychological pressure), were found to have higher scores in depression and chronic stress. This points again to the tremendous level of stress generated within the context of elite sports. Regarding burnout, numerous studies highlighted the connection between stress and symptoms of burnout (Raedeke and Smith, 2004; De Francisco et al., 2016; Frank et al., 2017).

Recent research indicated a number of potentially important factors (personal and environmental) associated with burnout and depression. In burnout research, such factors are even considered and examined in longitudinal studies in order to help evaluate etiological theory. However, in depression research, most studies are conducted in a cross-sectional manner and even less evidence is gathered for burnout and depression in a combined or comparable manner. Therefore, little knowledge is available to clearly point to certain factors which could help prevent both syndromes. In order to gather such evidence, a combined theoretical framework which can be applied for both constructs is proposed.

### Diathesis-Stress Model for Depression and Burnout

The development of depression is most often described by a diathesis-stress model (e.g., Haffel et al., 2005; Alloy et al., 2006; Hyde et al., 2008). Here certain vulnerabilities (genetics, social aspects, cognitive distortions, etc.) in combination with a stressor (chronic or acute) can lead to depression (Lee et al., 2010). Lee et al. (2010) highlighted the importance of stress with regard to depressive disorders in the general population in their review based on psychological and physiological evidence. In athletes, this connection to stress seems to be especially important due to the nature of elite sports and the aforementioned evidence showing connections between stress and depression in athletes (De Francisco et al., 2016).

When investigated simultaneously or compared, burnout and depression both can be described in a similar conceptual manner, strongly linked through the significance of stress on the development of both constructs (Frank et al., 2017). With regard to athlete burnout, Smith (1986) pointed out that burnout can be a potential outcome for athletes unable to efficiently cope with the chronic psychological stress involved in elite sports. More recently, Gustafsson et al. (2011) suggested an integrated burnout model which highlights the importance of stress with regard to athlete burnout.

Summing up the theoretical conceptions, both constructs (burnout and depression) are based on a temporal, stress-related process model assuming the development of either construct following unfortunate personal (dysfunctional attitudes, perfectionism, negative coping strategies) or environmental (cohesion) factors coexisting with severe stressors (i.e., chronic and acute stress). Integrating this knowledge, we propose a shared model for depression and burnout in athletes: a sport-specific diathesis-stress model. In this approach, we consider both burnout and depression in a diathesis-stress model, with shared sport-specific diathesis and stressors. In combination, important personal (e.g., cognition, attitudes, and strategies) and environmental factors (e.g., stressors) would lead to depressive syndromes, burnout or both. Further, we consider diathesis and stress factors specific to athletes and elite sports to account for the unique context of elite sports.

### Aim of the Study

Our aim with this study is to overcome methodological shortcomings by longitudinally assessing depression and burnout for one sport season. With this approach, we try to verify the validity of a diathesis-stress model within the sport context and try to uncover diatheses and stressors especially significant for elite athletes. Due to the severity of burnout and depression; the implications, harm and prejudice that still go along with this topic in elite sports; and the stigma associated with mental disorders (Steinfeldt and Steinfeldt, 2012; Bauman, 2016) we see a strong need for a preventative approach. Therefore, we aim to assess the data on a junior elite athlete sample.

Consequently, the present study addresses the research question of which diathesis and stressors show a significant impact on an increase of depression and burnout throughout one sport season. For this longitudinal analysis, we assessed possible vulnerabilities (lack of cohesion, coping strategies, perfectionism, attribution after failure, dysfunctional attitudes and athletic identity) in a training/preparation phase; the stressors (chronic stress, lack of recovery) were assessed in a competition phase and burnout and depression were assessed at the end of the season in a recovery phase. This procedure allows us to test the present diathesis and stressors in its proposed structure of a process model (diathesis-stress model) with the assumption of a temporal progression.

## Materials and Methods

### Participants

Participating junior elite athletes were recruited from a scientific project which was reviewed, ethically approved and financially supported by the German Federal Institute of Sport Science (*Bundesinstitut für Sportwissenschaft; BISp*) to investigate and help prevent depression and burnout in young elite athletes. The project had further been ethically approved by the dean of the department. The project’s goal was to enhance knowledge on burnout and depression. Gathered information should be transferred into recommendations for prevention. Participants were recruited through their sport associations, which were willing to participate in the project. Initial contact details were provided by officials from the participating sport associations or sport clubs. Participating athletes were contacted via email and additionally informed in an initial information meeting. Therefore, only junior athletes with high competition level (at least regional selection squad or members in professional junior development facilities) were included in the study. Data collection was carried out through an online platform. Participants had been send an e-mail at the specific predetermined dates with a link to the online questionnaire. At the first time of assessment (T1), a total of *N* = 194 German junior elite athletes (*M*_age_ = 15.08; *SD* = 1.95) participated in the study. Participants came from different sport disciplines: badminton (*n* = 9), gymnastics (*n* = 5), hockey (*n* = 15), ice running (*n* = 19), mountain bike (*n* = 16), short track (*n* = 12), soccer (*n* = 113) and swimming (*n* = 10). A total of *N* = 85 German junior elite athletes (*M*_age_ = 14.82; *SD* = 2.26) provided data on all three points of assessment and were therefore included in the prospective analysis.

### Measures

#### Depression

Depressive symptoms in junior athletes were assessed with the widely used German version of the Center for Epidemiologic Studies Depression Scale (CES-D) from the National Institute of Mental Health (Radloff, 1977; Hautzinger et al., 2011). The CES-D is a short self-report scale designed to measure depressive symptomatology in the general population. It was also repeatedly used to assess depressive symptoms among elite athletes (Yang et al., 2007; e.g., Armstrong and Oomen-Early, 2009; Junge and Feddermann-Demont, 2016). The 20 items are assessed on a scale ranging from 0 to 3, with higher scores indicating higher levels of depressive symptomatology. The scale is constructed, reliable and standardized for the age range 11–90 years. The scale has been found to have high internal consistency (α = 0.89), which in the present study was α = 0.85.

#### Burnout

Symptoms of burnout in athletes was assessed using the Athlete Burnout Questionnaire (ABQ; Raedeke and Smith, 2001) in its German version (Ziemainz et al., 2004). The ABQ is a self-report scale designed to assess the three core dimensions in athlete burnout: physical and emotional exhaustion, sport devaluation and reduced sense of accomplishment. The questionnaire consists of 15 items on a scale ranging from 0 to 3 with higher scores indicating greater levels of burnout symptomatology. There have been critiques due to missing relation of the three dimensions and it is recommended to evaluate the three dimensions independently (Gerber et al., 2018b). However, the scale is widely used for assessing burnout in sports and had previously be shown to be valid and reliable and also had acceptable internal consistency with Cronbach’s α = 0.86 in the present sample.

#### Chronic Stress

Chronic stress was assessed using the Screening of the Trier Inventory of Chronic Stress (TICS; Schulz et al., 2004). The scale is designed to measure experiences of chronic stress and therefore covers the last 3 months. The 12-item scale is a short self-report screening with items covering frequencies of experiences and feelings during the last 3 months. Answers are coded on a 5-point Likert-scale ranging from 0 (*never*) to 4 (*very often*). The scale was found to be valid and reliable (Schulz et al., 2004) in the general population. In German (junior) athletes, the scale was also validated and normed from the age of 16 years on with a reliability of Cronbach’s α = 0.87 (Hoffmann and Sallen, 2012). The scale has also been used in younger samples (Sallen et al., 2015) and showed good internal consistency in the present study with α = 0.90.

#### Recovery

Current state of recovery was assessed using a short self-description protocol adopted from the RESTQ-Sport (Kellmann and Kallus, 2001). The RESTQ-Sport is often used for assessment and monitoring of stress and recovery states among athletes (for review see Kellmann, 2010) and provides good psychometric properties (Kellmann and Kallus, 2001). The protocol uses the most important items in covering key aspects of the RESTQ-Sport (Kellmann, 2000). The questionnaire covers the previous 7 days and therefore provides a measure of an individual’s current state of recovery. The internal consistency of the used scale was acceptable with Cronbach’s α = 0.79.

#### Cohesion

Cohesion in team and individual athletes was measured using the German version of the Group Environment Questionnaire (GEQ; Carron et al., 1985) by Ohlert (2012). The GEQ is a widely used questionnaire to assess cohesion by four factors, namely group integration (related to task), group integration (social), individual attraction to group (related to task), and individual attraction to group (social). The widely used GEQ was translated, adapted and validated by (*N* = 418) German athletes (Ohlert, 2012). Adaption of the German version allowed assessment of cohesion in team and individual sports. We used 18 items with a nine-point Likert scale (strongly agree to strongly disagree). The scale was found to be internally consistent with Cronbach’s alpha ranging between α = 0.74 and α = 0.78 for the four subscales. Overall, reliability was good in the present study with an internal consistency of α = 0.81.

#### Perfectionism

Perception of perfectionistic expectations from outside was assessed using the subscale of the German Version of the Multidimensional Inventory of Perfectionism in Sport (MIPS; Stoeber et al., 2004). The MIPS was developed following existing questionnaires dominating research in the field of perfectionism (e.g., Frost Multidimensional Perfectionism Scale; FMPS; Frost et al., 1990; Multidimensional Perfectionism Scale; MPS; Hewitt and Flett, 1991b). The scale consists of nine subscales which can be regarded as either adaptive or maladaptive (Stoeber et al., 2004). In the present study we used the subscales *perception of perfectionistic expectations from outside* and *negative reactions after imperfection*, both of which are considered maladaptive (see Ashby and Rice, 2002; Enns and Cox, 2002). Both subscales have eight items on a 6-point Likert scale and its validity and reliability was tested in two studies indicating good internal consistency (study 1: Cronbach’s α ranging from 0.86 to 94). Reliability was good in the present study with Cronbach’s α = 0.88 for *perception of perfectionistic expectations from outside* and α = 0.89 for *negative reactions after imperfection*.

#### Attribution After Failure

Attribution after failure was assessed using the relevant dimensions internality, stability and globality after the most recent failure according to the Sport Attributional Style Scale (SASS; Hanrahan and Grove, 1990). Athletes had to rate the personal cause for failure and success on the following dimensions: internality, stability, globality, personal controllability, external controllability, and intentionality on separate 7-point bipolar scales, with higher scores indicating stronger attribution to the respective dimension. The SASS was shown to have adequate psychometric properties (Hanrahan and Grove, 1990). For analysis in the present study, the sum score for the three dimensions internality, stability and globality for the most recent failure was used.

#### Dysfunctional Attitudes

The Dysfunctional Attitudes Scale (DAS-A; Weissman and Beck, 1978) is a self-report scale designed to measure the presence and intensity of dysfunctional attitudes. The DAS assesses dysfunctional beliefs that are thought to reflect a person’s self-evaluation on 2 dimensions: perfectionism/performance evaluation and dependency. The original form (DAS-A) consists of 40 items and each items consists of a statement and a 7-point Likert scale (7 = *fully agree*; 1 = *fully disagree*). For economic and psychometric reasons, short version of this original scale has been proposed and validated, also in German (de Graaf et al., 2009; Rojas et al., 2015). In the present study we used the DAS-SF_1_ according to Beevers et al. (2007). The scale showed good internal consistency with Cronbach’s alpha = 0.84. Reliability was acceptable in the present study with an internal consistency of α = 0.74.

#### Coping Strategies

Athletes’ coping responses to life stressors were measured by using the stress-coping questionnaire (SVF; Erdmann and Janke, 2008). The SVF was also selected because of its focus on dispositional coping, rather than on situational coping, meaning that it indicates a temporally consistent coping style in the subject being tested. The questionnaire provides a comprehensive inventory of methods, allowing flexibility in individual procedures depending on the research question. In the case of elite athletes, Erdmann and Janke (2008) propose three negative strategies (escaping the situation, resignation, and self-pity). These coping strategies are assessed with 18 items, and answers are provided on a 5-point rating scale ranging from 0 to 4, with higher scores indicating more frequent use of this coping strategy. Higher scores on the respective coping strategies indicate a frequent and favored use. The SVF showed acceptable internal consistencies with Cronbach’s alpha ranging from α = 0.85 and α = 0.86 for the three subscales. This was confirmed for the present study with an internal consistency ranging between α = 0.82 and α = 0.83 for the respective subscales.

### Procedure

After review and approval for the project by the BISp, written informed consent by athletes and parents of each participating athlete was provided. Data was assessed pseudonymously (by a code derived from random numbers and letters for each participant) and pre-season in all sport disciplines with an online questionnaire battery. In case of interest or for further information on personal data, participants could use an individual code to access their individual data. Longitudinal data was assessed at specific, predetermined dates. Therefore, dates were adjusted to the seasonal schedule to each sport discipline by the trainers of participating teams. Times of assessment were: T1 preparation phase, T2 competition phase and T3 recovery phase. In order to account for the temporal progression and the assumptions of the diathesis-stress model, assessment had been according to the model. Variables considered as diatheses had been assessed at T1, assuming an already existing degree of the different diatheses. Variables considered to cover stressors were assessed at T2, aiming to capture a critical phase of competition at this time of assessment. We note, that stressors can be various and individual, thus they might be relevant at any time during the sporting season. However, assessment at T2 would fit the assumptions according to the diathesis-stress model the most. The dependent variables were assessed at T1 for baseline effects and at T3 to capture the changes after the course of the season.

### Analysis Procedure

Data was analyzed in two steps. First, for identifying relevant factors we conducted a variable selection using multiple linear regression analyses. Second, for testing diathesis and stressors, a hierarchical linear regression was performed. In both analyses, we followed a stepwise approach. The first step was the inclusion of depression, respectively, burnout, at T1 for controlling initial differences in the dependent variable. Furthermore, gender, sport discipline and socioeconomic status (SES) were included in step 1, also controlling for initial effects of these relevant factors, possibly influencing baseline effects. As second step, we included diathesis factors measured at T1. We considered the following diatheses for variable selection: dysfunctional attitudes, coping resignation, coping flight, coping self-pity, coping positive self-instruction, cohesion, negative attribution after failure, perfectionistic expectations from outside and negative reactions to imperfection. The third step was the inclusion of the stressor factors (chronic stress and recovery) at T2. Burnout and depression (measured at T3) were included as dependent variables and for each dependent variable we computed a linear model.

Variable selection for identification of relevant factors was based on the Akaike’s information criterion (AIC) by backwards selection. Therefore, all possible factors in a step were included in the regression model and excluded based on a drop in AIC values. Hierarchical model testing was based on these three steps as well. Thus, the model resulting from step 1 was compared to the model resulting from step 2, and the model resulting from step 2 was compared to the model resulting from step 3. Model comparison consisted of a Chi-Square test on an alpha level of 0.05. Notably, the compared models only included factors identified by the variable selection. It was important to minimize the number of factors in the regression models to account for the relatively low sample size.

## Results

In the present sample, mean scores for depressive symptoms were *M* = 10.52; *SD* = 7.10. For burnout symptoms, mean scores were *M* = 16.60; *SD* = 8.03 for T1. At T3 mean scores for depressive symptoms were *M* = 10.76; *SD* = 8.43 and *M* = 16.91; *SD* = 8.98 for burnout symptoms.

From the original *n* = 194 junior elite athletes recruited at T1, only *n* = 85 completed all three assessment times. No significant differences between non-completers and completers were observed at T1 [for burnout: *t*(182) = 0.73, *p* = 0.469; for depression: *t*(190) = 1.23, *p* = 0.219]. Also, comparisons of predictors between non-completers and completers revealed no significant *t*-test. Therefore, missing data was accounted for by list-wise deletion following Graham (2009).

### Variable Selection

In the linear models considering depression as the dependent variable, step one in variable selection indicated the best model fit by removing the factor gender (drop in AIC = 1.84), and SES (drop in AIC = 0.54). The factor sport discipline was removed in the second step, resulting in a drop in AIC of 1.98. Only the initial value in depressive symptoms (T1) remained in the model (*b*^∗^ = 0.39; *p* < 0.01). In step 2, the factors perfectionism (pressure from outside) with a drop in AIC = 1.99, negative attribution after failure (drop in AIC = 1.99), positive coping strategy self-instruction (drop in AIC = 1.72), self-pity (drop in AIC = 1.02), and cohesion (drop in AIC = 0.67) were removed from the regression model. In step 3, the factors athlete burnout (drop in AIC = 1.30), and chronic stress (drop in AIC = 0.3) were removed.

When burnout was tested as the dependent variable, step 1 indicated the best model fit by removing the factor SES (drop in AIC = 1.97), and gender (drop in AIC = 1.86). The factor sport discipline was removed afterwards resulting in a drop in AIC of 1.66. Only the initial value in athlete burnout (T1) remained in the model; however, it was a non-significant predictor (*b*^∗^ = 0.20; *p* = 0.128). In step 2, the factors cohesion (drop in AIC = 1.68), negative coping strategy flight (drop in AIC = 1.60), positive coping strategy self-instruction (drop in AIC = 1.49), negative attribution after failure (drop in AIC = 0.79) had been removed. The factors negative coping strategy self-pity (drop in AIC = 1.39), and perfectionism (pressure from outside) with a drop in AIC = 0.09 were removed from the regression model in the third step. The remaining factors for both models (burnout and depression) can be seen in Tables 1, 2.

**TABLE 1:** Hierarchical multiple linear regression results regarding depressive symptoms.

**Step**	**Factor**	**Estimator**	**Model**	**Model comparison**
1	Depression (T1)	b* = 0.30**	*R*^2^ = 0.30 *F* = 36.25; *p* < 0.001 RSS = 4145	
2	Dysf. attitudes	b* = 0.27*	*R*^2^ = 0.42	ΔRSS = 683
	Perfectionism	b* = −0.17 n.s.	*F* = 11.37; *p* < 0.001	*F* = 3.89; *p* < 0.01
	Coping: resignation	b* = 0.34*		
	Coping: flight	b* = −0.24 n.s.	RSS = 3472	
3	Recovery	b* = −0.28**	*R*^2^ = 0.47	ΔRSS = 336
			*F* = 11.75; *p* < 0.001	*F* = 8.35; *p* < 0.01
			RSS = 3136	

* *p* < 0.05; ** *p* < 0.01.

**TABLE 2:** Hierarchical multiple linear regression results regarding burnout.

**Step**	**Factor**	**Estimator**	**Model**	**Model comparison**
1	Burnout (T1)	b* = 0.18 n.s.	*R*^2^ = 0.38	
			*F* = 50.8; *p* < 0.001	
			RSS = 4206	
2	Dysf. attitudes	b* = 0.30**	*R*^2^ = 0.53	ΔRSS = 1050
	Perfectionism	b* = −0.19*	*F* = 23.0; *p* < 0.001	Δ*F* = 8.88; *p* < 0.001
	Coping: resignation	b* = 0.27*	RSS = 3156	
3	Chronic stress	b* = 0.22*	*R*^2^ = 0.58	ΔRSS = 327
	Recovery	b* = −0.13 n.s.	*F* = 18.2; *p* < 0.001	Δ*F* = 4.51; *p* < 0.05
			RSS = 2829	

* *p* < 0.05; ** *p* < 0.01.

### Hierarchical Model Testing

Comparison of the regression models with depression as the dependent variable showed a significantly better model fit for the step 2 model with psychological diathesis over the initial step 1 model from (*F* = 3.89; *p* < 0.01). Further, the step 3 model, which included stressors resulted in a significant improvement over the step 2 model (*F* = 8.35; *p* < 0.01). Further inclusion of plausible interactions did not reveal any significant value. The interaction between dysfunctional attitudes and recovery was not significant, with *F* = 3.55; *p* = 0.063. Further, including the interaction between the coping strategy resignation and recovery did not improve the model (*F* = 0.15; *p* = 0.697).

Comparison of the regression models with burnout as the dependent variable showed a significantly better model fit for the model with the psychological diathesis (step 2) over the initial model from step 1 (*F* = 8.88; *p* < 0.001), Furthermore, the step 3 model, which included stressors significantly improved the model in step 2 (*F* = 4.51; *p* < 0.05). Further inclusion of plausible interactions did not reveal any significant improvement. Inclusion of the interaction between dysfunctional attitudes and chronic stress did not improve the model (*F* = 0.17; *p* = 0.895). The inclusion of the interaction between the coping strategy resignation and chronic stress also did not improve the model (*F* = 2.36; *p* = 0.128).

*Post hoc* power analysis for the regression models was performed using Gpower (Erdfelder et al., 1996). For both models, the effect size was calculated based on increase in *R*^2^ from the base model, which already included the dependent variable at the first time of assessment (T1) to account for baseline differences. For burnout, the effect size was *f*^2^ = 0.25 and reached with a sample size of *N* = 85 power of 0.95. For depression, the effect size was somewhat smaller with *f*^2^ = 0.20 leading to a power of 0.89.

## Discussion

Most research on depression and burnout in elite athletes is cross-sectional and correlational in nature. There exists strong need for a longitudinal assessment of depression (at least one sporting season) in order to test the validity of a diathesis-stress model in elite sports and to identify vulnerabilities that increase the risk of developing a depression or burnout. In its theoretical conception, depression and burnout are explained by a temporal, stress-related process model. This model assumes the development of depression and burnout from unfortunate factors, whether personal (e.g., dysfunctional attitudes, perfectionism, negative coping strategies) or environmental (e.g., conflicts in teams), which interact with severe stressors (chronic stress). The goal of the present study was to longitudinally assess vulnerabilities and stressors regarding symptoms of depression and burnout throughout one sporting season. The data supports the validity of a diathesis-stress model and identifies the following factors as relevant diatheses to burnout and depression in elite athletes: dysfunctional attitudes, negative coping strategy of resignation, missing recovery (depression only) and high levels of chronic stress (for burnout). The structure of a process model (diathesis-stress model) can be concluded with the assumption of a temporal progression.

**FIGURE 1:**
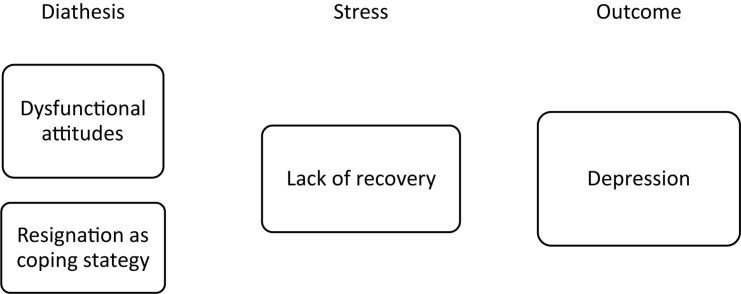
Sport-specific diathesis stress model for depression.

**FIGURE 2:**
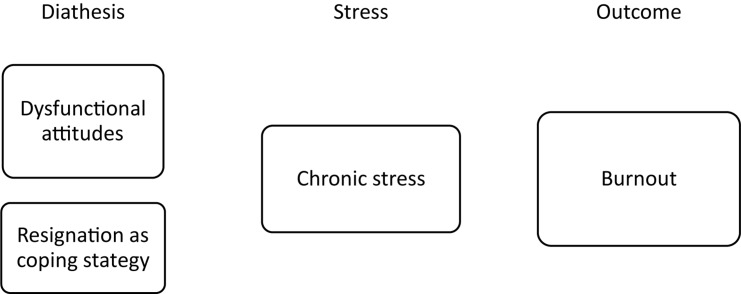
Sport-specific diathesis stress model for burnout.

A longitudinal study identifying psychological vulnerabilities offers the additional benefit of elucidating the mechanisms underlying these relevant psychological syndromes. Promoting mental health in athletes is an important issue that has only recently been addressed more urgently (Schinke et al., 2017). An understanding of the mechanisms and etiologies underlying mental health issues in elite sports is necessary to develop and improve athlete-centered prevention and treatment programs. Our results deliver insights into such mechanisms regarding the syndromes of depression and burnout. They provide a chance to draw a first draft of a sport-specific diathesis-stress model for depression and burnout separately. Figures 1, 2 present the psychological diathesis incorporated in a sport-specific diathesis-stress model. Previous research on depression and burnout has indicated that sport specific mechanisms should be considered. In the case of depression, differences in sport-specific variables such as sport discipline (Nixdorf et al., 2016) or the presence of an upcoming championship (Hammond et al., 2013) highlighted the unique context of elite sports. In case of burnout, the findings on the importance of support by teammates (DeFreese and Smith, 2013) or the relation to overtraining (Lemyre et al., 2007) might represent such sport specificities. Thus, a deeper understanding for sport-specific mechanisms would certainly improve overall knowledge of both of these syndromes. The introduced sport-specific diathesis-stress model can function as a general framework for further elaboration and used for research to test assumptions of the genesis of burnout and depression. Further research might therefore identify other important variables or test more specific hypotheses on the interaction of certain diathesis and stressors.

Regarding both constructs, dysfunctional attitudes and resignation as a negative coping strategy appear as important predictors for increases in symptomatology. Thus, these factors should be further considered and investigated in athletes. In terms of dysfunctional attitudes, its relation for perfectionism has already been discussed, but mainly in research relating to the general population (Ashby and Rice, 2002; Beevers et al., 2007). Sports research predominantly focuses on perfectionism, and some researchers argue that perfectionism also has adaptive components (Gotwals et al., 2012). The present findings, however, indicate that with regard to mental health and the understanding of clinical syndromes, dysfunctional attitudes might serve as a hint to distinguish adaptive from maladaptive cognition in perfectionistic striving. In regards to negative coping strategies, previous research highlighted connections to depression (Nixdorf et al., 2013) and burnout (Raedeke and Smith, 2004). The present findings underpin the role of coping with stress in elite sports. Considering that stress and a lack of recovery presented as predictive stressors, the importance of coping mechanisms becomes even more apparent.

With the knowledge of important diathesis and their temporal, developmental link, prevention can become athlete-specific, effective and economical. Romano and Hage (2000) define prevention as including one or more of the following: (1) stopping a problem behavior from ever occurring; (2) delaying the onset of a problem behavior, especially for those at-risk for the problem; (3) reducing the impact of a problem behavior; (4) strengthening knowledge, attitudes, and behaviors that promote emotional and physical well-being; and (5) promoting institutional, community, and government policies that further physical, social, and emotional well-being of the larger community. This conceptualization is consistent with Caplan (1964) who identified prevention interventions as primary, secondary, and tertiary prevention, and with the alternative definition by Gordon (1983) that identified prevention interventions as universal, selected, and indicated for those not at risk, at risk, and experiencing early signs of problems. Taking into account the significant aforementioned psychological vulnerabilities, such as dysfunctional attitudes and negative coping for depression and for burnout, we can now, empirically reason for interventions specifically targeting these vulnerabilities. The goal here should be to actively shape primary prevention and increase individual resources regarding, for example, recovery. Romano and Hage (2000) also mention that although dimensions 1, 2, and 3 can be conceptualized in traditional primary, secondary, and tertiary terms and refer to the individual, dimensions 4 and 5 are conceptualized within a “risk-reduction” -framework. Regarding the community of elite sports this definition on an institutional level seems adequate and necessary. Therefore, prevention of mental disorders should not only be seen as an individual challenge, but as an organizational problem warranting organizational solutions as well. To overcome the stigma of mental disorders in elite sports (Gulliver et al., 2012) on a larger scale, changes in social climate might be necessary. However, first steps might root in solid information and offers of support for athletes and coaches; leading to a general prevention of mental disorders. Providing information on the relevance (e.g., prevalence rates), the syndrome itself and the possibilities for further information and help is therefore important. These steps would fall into dimensions 4 and 5 of the above mentioned definition by Romano and Hage (2000). However, it might be even more important not just to inform on mental health but to provide information on where athletes can seek help. It would be misdirected, to impose feelings of responsibility onto coaches and officials, but providing access to practitioners or initiatives with expertise in prevention and treatment of burnout and depression is an important task.

## Limitations

The present study is longitudinal in nature. While this allows a temporal analysis of the dependent variables, with causal implications at the end, participant attrition is common. In this study, the dropout rate of 56% for data assessment over 1 year is unfortunate, but appears to be average and still offers statistical significance with suitable power (Green, 1991). For the linear regression models, the present sample size showed acceptable power. However, it should be noted that variables with only small effects might have been undiscovered due to the sample size. As mentioned earlier, no differences on the dependent variables were found between athletes who participated at all 3 times of assessment compared to those who dropped out of the study. This improves validity of the found results to some degree. Still, the sample size is relatively low and, according to the guideline from Green (1991), the present sample size would be sufficient for the model comparison, but beta weights should be interpreted with caution.

As mentioned above, the present study assessed burnout and depressive symptoms in a relatively young sample. Previous studies have been concerned with assessing the prevalence rates and approached a categorization of, for example, depressed vs. non-depressed athletes (Schaal et al., 2011; e.g., Gouttebarge et al., 2016; Junge and Feddermann-Demont, 2016). Despite the clinical construct of depression, the present study assesses syndromes only on a symptom-based level and offers no clinical diagnosis. As the goal is to identify predictive factors, we focused on increases in the level of symptomatology, not on a clinical syndrome. The investigated changes in symptomatology might therefore not reflect any significant increases. Therefore, assumptions on the clinical relevance of this effect require further exploration.

The clinical relevance of burnout remains unclear as well. This is due to a lack of consensus in the broader scientific community on the measurement and diagnosis of burnout (Bianchi et al., 2015a), but also due to the missing strength in assessment. In the present study we used the ABQ, which is the common instrument in sports (Gustafsson et al., 2017). However, the ABQ had been critiqued because of low inter-correlations and missing relatedness of the three dimensions over time (Gerber et al., 2018b; Lundkvist et al., 2018). In the present study, we used the composite score, as the main goal was to find predictors on a general scale and compare these to depression. The composite score has previously been used (Frank et al., 2017), but taking into consideration the potential heterogeneity of burnout symptoms over time (Martinent et al., 2016), further research should as well investigate more closely the validity of our findings for each of the three dimensions. Further, findings on possible interplay between the dimensions should be considered as well (Isoard-Gautheu et al., 2015). In addition, the challenge of defining burnout remains and further research has to work on a solution on this issue.

## Conclusion

In a sample of junior elite athletes, the present study confirms the structure of a diathesis-stress model with the assumption of temporal progression for burnout and depression symptoms in elite athletes. Overlapping vulnerabilities for both outcomes were identified: dysfunctional attitudes and the negative coping strategy of resignation. However, stressors appear to be different for depression (missing recovery) and burnout (high levels of chronic stress). Perfectionism did not have a significant effect on the development of depression, but it did show a significant, but contrary to hypothesis, effect on burnout. The goal of the study was to uncover underlying mechanisms connected to sport in order to support prevention for athlete populations. Practitioners could utilize these findings by considering the aforementioned vulnerabilities and stressors in junior elite athletes in order to prevent negative outcomes such as burnout and depression.

## Data Availability Statement

The datasets generated for this study are available on request to the corresponding author.

## Ethics Statement

Ethical review and approval was not required for the study on human participants in accordance with the local legislation and institutional requirements. Written informed consent to participate in this study was provided by the participants’ legal guardian/next of kin.

## Author Contributions

The first research idea and proposal for this study was written by all three parties together. IN and RN were trusted with the data assessment and data analysis. All authors contributed to the manuscript in equal terms.

## Conflict of Interest

The authors declare that the research was conducted in the absence of any commercial or financial relationships that could be construed as a potential conflict of interest.
